# The delta opioid receptor agonist KNT‐127 relieves innate anxiety‐like behavior in mice by suppressing transmission from the prelimbic cortex to basolateral amygdala

**DOI:** 10.1002/npr2.12406

**Published:** 2023-12-29

**Authors:** Ayako Kawaminami, Daisuke Yamada, Toshinori Yoshioka, Azumi Hatakeyama, Moeno Nishida, Keita Kajino, Tsuyoshi Saitoh, Hiroshi Nagase, Akiyoshi Saitoh

**Affiliations:** ^1^ Laboratory of Pharmacology, Department of Pharmacy, Faculty of Pharmaceutical Sciences Tokyo University of Science Noda Japan; ^2^ International Institute for Integrative Sleep Medicine (WPI‐IIIS) Tsukuba Japan; ^3^ University of Tsukuba Tsukuba Japan

**Keywords:** anxiety, basic, basolateral amygdala, delta opioid receptor, excitatory synaptic transmission, prelimbic cortex

## Abstract

**Aim:**

Excitatory projections from the prelimbic cortex (PL) to the basolateral nucleus of the amygdala (BLA) are implicated in the regulation of anxiety‐like behaviors, and we previously demonstrated that anxiolytic‐like effects of the selective delta‐opioid receptor (DOP) agonist KNT‐127 is involved in suppressing glutamate neurotransmission in the PL. Here, we investigated the mechanisms underlying the anxiolytic‐like effect of KNT‐127 in mice by combining optogenetic stimulation of the PL–BLA pathway with behavioral analyses.

**Methods:**

Four‐week‐old male C57BL/6J mice received bilateral administration of adeno‐associated virus (AAV)2‐CaMKIIa‐hChR2(H134R)‐enhanced yellow fluorescent protein (EYFP) into the PL to induce expression of the light‐activated excitatory ionic channel ChR2. Subsequently, an optic fiber cannula connected to a wireless photo‐stimulator was implanted into the BLA for optogenetic PL–BLA pathway stimulation. We evaluated innate anxiety using the elevated plus maze (EPM) and open field (OF) tests as well as learned anxiety using the contextual fear conditioning (CFC) test.

**Results:**

Optogenetic activation of the PL–BLA pathway enhanced anxiety‐like behaviors in the EPM and OF, while prior subcutaneous administration of KNT‐127 (10 mg/kg) reduced this anxiogenic effect. In contrast, optogenetic activation of the PL–BLA pathway had no significant effect on conditioned fear.

**Conclusion:**

Our findings indicate that the PL–BLA circuit contributes to innate anxiety and that the anxiolytic‐like effects of KNT‐127 are mediated at least in part by suppression of PL–BLA transmission. The PL delta‐opioid receptor may thus be an effective therapeutic target for anxiety disorders.

## Abstract 2

Our findings indicate that the PL–BLA circuit contributes to innate anxiety. In Addition, the anxiolytic‐like effects of KNT‐127 are mediated PL–BLA circuit.

## Graphical abstract

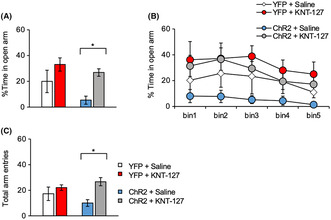

## 1 INTRODUCTION

The delta opioid receptor (DOP) is predominantly localized in brain regions associated with emotional regulation and has been implicated in the modulation of anxiety‐related behaviors.^1^, ^2^, ^3^ Notably, DOP agonists have demonstrated potent anxiolytic‐like effects in rodents, warranting further exploration of the underlying mechanisms to aid in drug development for anxiety disorders.^4^, ^5^, ^6^, ^7^

Previous studies utilizing in vivo reverse microdialysis have revealed that local perfusion of the voltage‐gated sodium channel activator veratrine within the mouse PL increases extracellular glutamate concentration and induces anxiety‐like behavior.^8^ Intriguingly, the selective DOP agonist KNT‐127 has been shown to attenuate the excessive glutamate release induced by veratrine and to exert anxiolytic‐like effects.^6^ Additionally, intra‐PL perfusion of KNT‐127 significantly reduced the number of cells expressing c‐Fos, a marker of neuronal activity, in PL projection sites such as the basolateral nucleus of the amygdala (BLA), and this effect was reversed by infusion of an N‐methyl‐D‐aspartic acid (NMDA) receptor inhibitor.^6^ Moreover, KNT‐127 inhibited glutamatergic neurotransmission in ex vivo PL slices by suppressing glutamate release from presynapses via DOP.^9^ These findings suggest that DOP activation may exert anxiolytic‐like effects by suppressing glutamate neurotransmission within the PL or from the PL to BLA.

In the current study, we examined the effects of PL–BLA neural projections on innate anxiety‐like behavior in mice by combining targeted optogenetic pathway activation with behavioral analyses using the elevated plus maze (EPM) and open field (OF) tests. Optogenetic pathway activation was also used to examine effects of PL–BLA neural projections on learned fear in the contextual fear conditioning (CFC) test. Additionally, we investigated the efficacy of the DOP agonist KNT‐127 to reduce anxiety‐like behaviors evoked by PL–BLA neural pathway activation.

## 2 MATERIALS AND METHODS

### 2.1 Animals

Male C57BL/6J mice (Japan SLC, 4 weeks old) were used and housed under controlled temperature (23 ± 1°C) and a 12‐h–12‐h light–dark cycle (lights on at 8:00 am) with unrestricted access to food and water.

### 2.2 Viral injection surgery

Mice were bilaterally administered with adeno‐associated virus (AAV) encoding channelrhodopsin 2 (ChR2) fused with enhanced yellow fluorescent protein (EYFP) under control of the CaMKIIa promoter (AAV2‐CaMKIIa‐ChR2[H134R]‐EYFP, obtained from Vector Core, University of North Carolina, NC, USA) or control AAV vector (AAV2‐CaMKIIa‐EYFP) into the PL (coordinates: AP: +1.65 mm, ML: ±0.4 mm, DV: −2.25 mm from the bregma).^10^ The AAV vectors were performed using 10 μL gas‐tight syringes (1701RN; Hamilton Company) at 0.2 μL/side and 0.1 μL/min using Ultra Micro Pump III (World Precision Instruments, Inc.).

### 2.3 Optical fiber implantation and optogenetic stimulation

Five weeks after the viral injection, a dual‐light‐emitting diode (LED) optic cannula (TeleLCD‐B‐5‐500‐6.2; BRC Bioresearch) was implanted into the BLA (coordinates: AP: −1.46 mm, ML: ±3.1 mm, DV: −4.3 mm from the bregma). Mice were monitored for at least 5 days postsurgery to ensure recovery, as evidenced by normal eating, drinking, and defecation. The PL–BLA neural circuit was activated specifically for behavioral analysis using a wireless optogenetic stimulation system (Teleopto, BRC Bioresearch Hashima), allowing the delivery of digital pulses (pulse width: 100 ms, pulse interval: 100 μs, pulse frequency: 10 Hz) via a stimulator (SEN‐7203, NIHON KOHDEN) to activate an implanted LED. A week later, each behavioral test was conducted. At the end of each test, the injection sites were verified, and subjects in which the position was inappropriate were discarded from the data.

### 2.4 Assessment of innate and conditioned anxiety‐like behaviors

#### 2.4.1 Elevated plus‐maze test (EPM test)

The EPM test was conducted over a 10‐min period. The EPM apparatus (LabDesign) consisted of four arms set in a cross pattern from a neutral central square. Vertical walls (closed arms, 25 cm × 6 cm × 30 cm) delimited two opposite arms, whereas the two other opposite arms had unprotected edges (open arms, 25 cm × 6 cm). The total number of entries into the closed and open arms as well as the cumulative time spent in the open arms (percent time in open arms) were quantified during a 5‐min observation window. An arm visit was recorded when a mouse extended at least half of its body into a given arm.

#### 2.4.2 Open‐field test (OF test)

Animals were monitored for 10 min while exploring an OF. The OF apparatus (MELQUEST Co) was a square arena (45 cm × 45 cm) subdivided into five equal parts to form one section (9 cm^2^) each, including a central area (27 cm × 27 cm). Behavioral measures included the proportion of time (%) spent in the center area (percent center time) and the number of crossing counts.^11^

#### 2.4.3 Contextual fear conditioning test (CFC test)

On the conditioning day (Day 1), mice received eight 0.8‐mA foot shocks (1 s in duration at 30 s intervals) by shock generator (ENV‐414; Med Associates) in the conditioning chamber (20 cm × 20 cm × 33 cm; LabDesign). After a 24‐h later (Day 2), mice were placed back into the same chamber without foot‐shock for 6 min. Freezing behavior was manually analyzed based on previous reports,^12^ during each session, by training the experimenter.

### 2.5 Drugs

KNT‐127 was synthesized at the University of Tsukuba and dissolved in saline immediately prior to subcutaneous (s.c.) administration at 10 mg/kg. Administrations were performed 30 min before the indicated EPM test.

### 2.6 Statistical analysis

Data are presented as mean ± standard error of the mean (SEM). The results of two groups were compared using Student's *t*‐tests and those for more than two groups using one‐ or two‐way analysis of variance (ANOVA) followed by Bonferroni's test for multiple comparisons. All analyses were conducted using GraphPad Prism 7 (GraphPad Software) with statistical significance set at *p* < 0.05.

## 3 RESULTS

**FIGURE 1:**
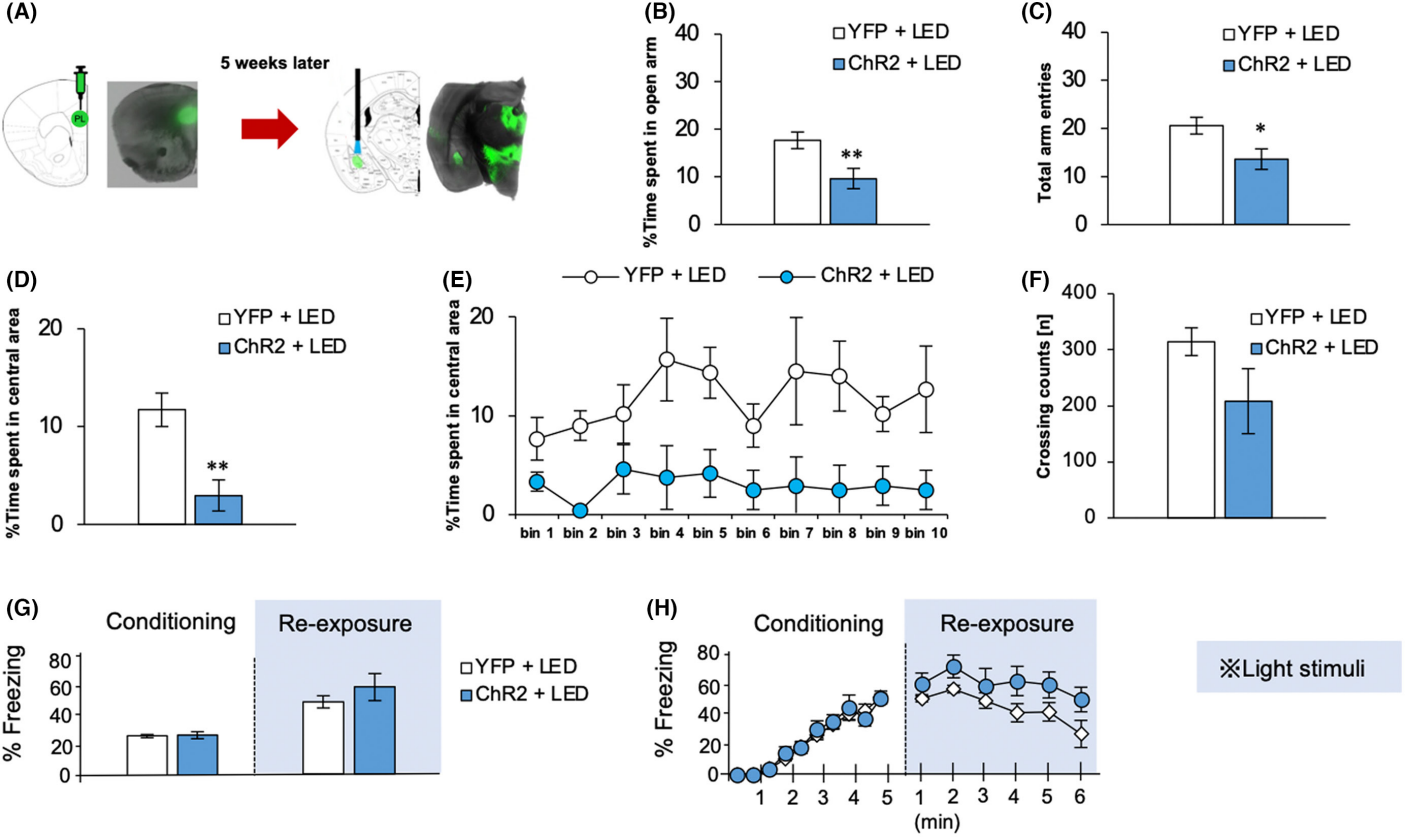
Experimental set‐up and behavioral analysis. (A) Schematic of the experimental procedures showing bilateral administration of adeno‐associated virus (AAV)2‐CaMKIIa‐hChR2(H134R)‐enhanced yellow fluorescent protein (EYFP) or AAV2‐CaMKIIa‐EYFP into the prelimbic cortex (PL) and implantation of a dual‐LED optic cannula into the basolateral amygdala (BLA) of 5‐week‐old mice. Parts of images were extracted from the mouse brain atlas by Paxinos and Franklin.^10^ (B, C) Effects of optogenetic PL activation on innate anxiety‐like behavior in the elevated plus maze (EPM) test. The yellow fluorescent protein (YFP) + LED group (*n* = 9) was injected with AAV2 vector encoding only YFP as the control treatment and stimulated by LED to activate the PL, while the ChR2 + LED group (*n* = 9) was injected with AAV2 vector expressing the light‐activated excitatory ionic channel ChR2 to induce PL excitation upon LED stimulation. (B) Percentage of time spent on the open arms. (C) Total arm entries. (D–F) Effects of optogenetic PL activation on innate anxiety‐like behavior in the open field (OF) test. The number of mice in each group was as follows: *n* = 12 for YFP + LED and *n* = 8 for ChR2 + LED. (D) Percentage of time mice spent in a central area. (E) Duration of central area exploration. (F) Number of crossing counts into adjacent areas. (G, H) Effects of optogenetic PL activation on conditioned anxiety‐like behavior in the contextual fear conditioning (CFC) test. The number of mice in each group was as follows: *n* = 12 for YFP + LED and *n* = 8 for ChR2 + LED. (G) Percent freezing on conditioning days (pairing of shock and context) and re‐exposure days (re‐exposure to context alone). (H) Time course of percent freezing in mice. Results are expressed as the mean ± standard error of the mean (SEM). *p < 0.05 and **p < 0.01 by unpaired Student's *t*‐test.

To investigate the contributions of the PL–BLA pathway to innate and conditioned anxiety‐like behaviors, mice expressing the light‐activated excitatory ionic channel ChR2 or YFP, specifically in the PL–BLA pathway, were examined in behavioral tests during optical stimulation in the test session or re‐exposure session (Figure 1A). We first conducted the EPM test. The % time in open arms (*p* = 0.0094; Figure 1B) and the number of arm entries (*p* = 0.035; Figure 1C) in the ChR2 group was significantly decreased compared with the YFP group. Moreover, the ChR2 group displayed a significant decrease in percent center time during the OF test (*p* = 0.0029; Figure 1D,E), also consistent with greater innate anxiety, while motor activity as measured by line crossings into other OF sections did not differ significantly between groups (Figure 1F). In contrast to innate anxiety, conditioned fear as measured by % freezing in the CFC remained comparable between ChR2 and YFP groups during stimulation (Figure 1G,H).

**FIGURE 2:**
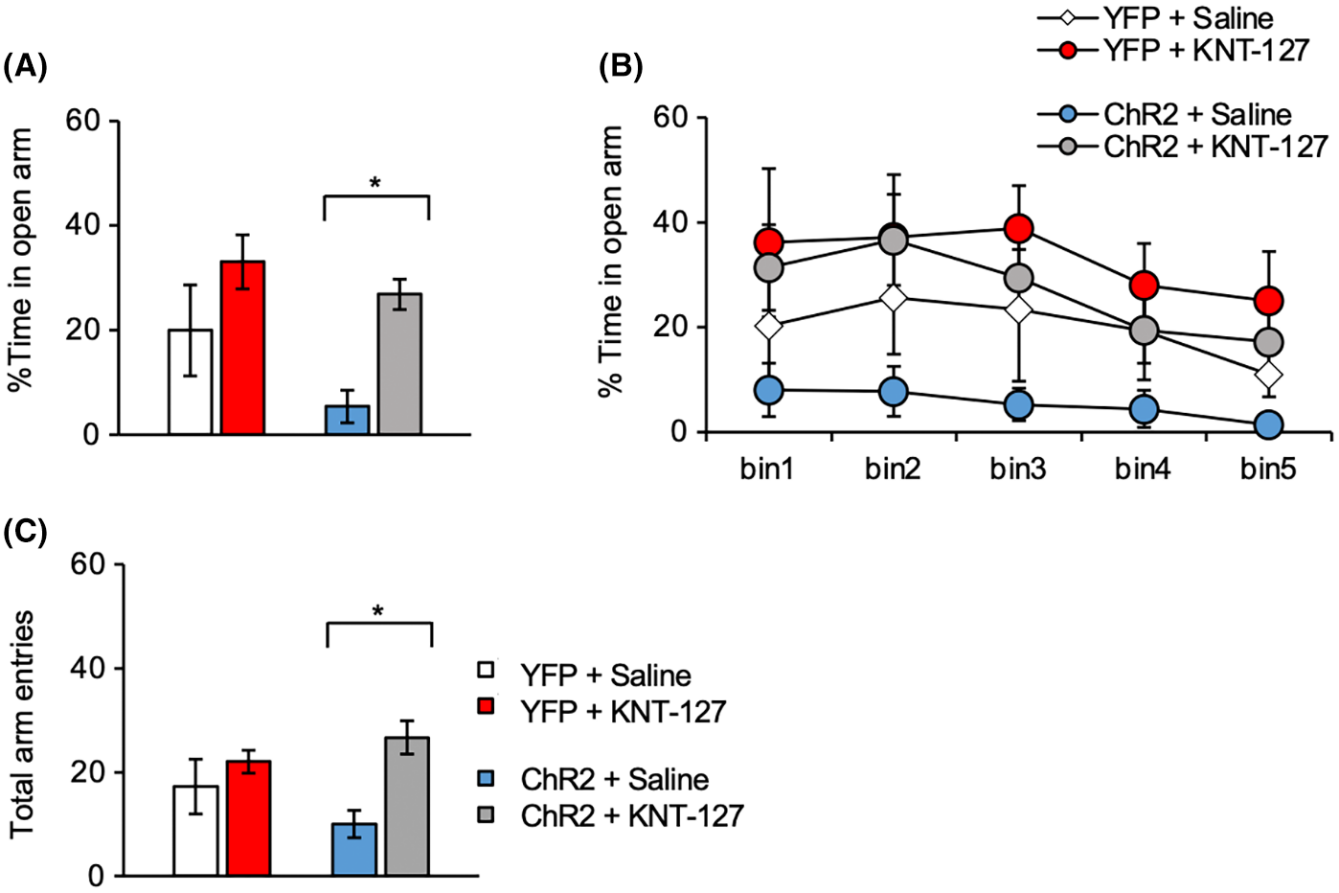
Modulatory effects of KNT‐127 on anxiety‐like behavior in the elevated plus maze (EPM) test. Mice received either saline or KNT‐127 (10 mg/kg, s.c.) 30 min before the test and 10‐Hz photo‐stimulation of the prelimbic cortex (PL)–basolateral amygdala (BLA) pathway during the test. (A) Percent time spent in the open arms. (B) Time course of percent time spent in the open arms. (C) Total arm entries. Results are expressed as the mean ± standard error of the mean (SEM). The number of mice in each group was as follows: *n* = 5 for the yellow fluorescent protein (YFP) + saline, *n* = 6 for YFP + KNT‐127, *n* = 6 for ChR2 + saline, and *n* = 6 for ChR2 + KNT‐127. *p < 0.05 by one‐way or two‐way ANOVA with post‐hoc Bonferroni tests.

Finally, we examined the effect of KNT‐127 on anxiety‐like behaviors induced by specific activation of the PL‐BLA pathway in the EPM test. The ChR2 + KNT‐127 group displayed increased percent time in open arms compared with the ChR2 + Saline group (*p* = 0.0413; Figure 2A). Similar effects were obtained in the time course of percent time spent in the open arms (Figure 2B). In contrast, there were no significant differences in these behavioral measures between YFP + KNT‐127 and YFP + saline groups (*p* = 0.0112; Figure 2C). These findings suggest that KNT‐127 mitigates innate anxiety‐like behavior resulting from specific PL–BLA pathway activation.

## 4 DISCUSSION

Specific activation of the PL–BLA pathway enhanced innate anxiety‐like behaviors in mice but had no detectable effect on contextually conditioned fear to context. In addition, KNT‐127 effectively reduced the innate anxiety‐like behavior induced by PL–BLA circuit activation.

Activation of PL has been reported to contribute to the induction of anxiety‐like behaviors.^13^, ^14^ More recent reports have also implicated the PL–BLA pathway, specifically in anxiety‐related processes.^15^ Our findings substantiate these prior observations, as specific activation of the PL–BLA pathway in mice consistently induced innate anxiety‐like behaviors.

As aforementioned, we demonstrated that KNT‐127 suppresses the increase in extracellular glutamate concentration in PLs via DOPs and simultaneously improves the anxiety‐like behavior of mice in a microdialysis study.^6^ Additionally, KNT‐127 significantly reduced the frequency of spontaneous miniature excitatory postsynaptic potentials (mEPSCs) in the PL and enhanced the paired‐pulse ratios of electrically evoked EPSCs.^9^ Collectively, these previous results and our combined optogenetic and behavioral experiments strongly suggest that KNT‐127 suppresses innate anxiety‐like behavior by inhibiting glutamate release and neuronal excitability in the mouse PL.

Specific activation of the PL–BLA pathway had no significant impact on conditioned fear‐like behaviors in the CFC test. This result contradicts reports that PL activation and PL‐BLA neural circuits are involved in the expression of conditioned fear.^16^, ^17^, ^18^ However, these reports focused on auditory fear, rather than contextual fear, which we examined in this study. These contradictions suggest that the PL‐BLA neural circuits may play different roles in contextual‐ and auditory‐conditioned fear. These results indicate that distinct neural pathways govern innate anxiety‐like behaviors and conditioned fear responses, of which the PL–BLA neural circuit is predominantly involved in innate fear expression. Future investigations on the detailed molecular, neurocellular, and circuit mechanisms are warranted to aid in the development of anxiolytic treatments targeting DOP signaling in the PL–BLA pathway.

## 5 CONCLUSION

This study provides compelling evidence that the PL–BLA pathway is a pivotal regulator of innate anxiety and a target for DOP‐induced anxiolytic effects.

## AUTHOR CONTRIBUTIONS

Ayako Kawaminami conducted the experiments and analyzed the data. Ayako Kawaminami, Toshinori Yoshioka, Daisuke Yamada, and Akiyoshi Saitoh wrote the manuscript. Moeno Nishida and Azumi Hatakeyama participated in data acquisition. Keita Kajino and Hiroshi Nagase synthesized and provided resources. Daisuke Yamada and Akiyoshi Saitoh designed and supervised the project. All authors discussed the results and contributed to manuscript refinement.

## FUNDING INFORMATION

This research was partially funded by the Cyclic Innovation for Clinical Empowerment from the Japan Agency for Medical Research and Development (AMED), grant number JP17pc0101018, awarded to AS.

## CONFLICT OF INTEREST STATEMENT

The authors declare no conflict of interest.

## ETHICS STATEMENT

Approval of the Research Protocol by an Institutional Reviewer Board: N/A.

Informed Consent: N/A.

Registry and the Registration No. of the study/trial: N/A.

Animal studies: The animal study was reviewed and approved by the Institutional Animal Care and Use Committee of the Tokyo University of Science (approval Nos. Y20020, Y21002, and Y22014).

## Supporting information


Data S1.

Data S2.

Data S3.

Data S4.

## Data Availability

The raw data that support the present results are available in the [Link], [Link], [Link], [Link].
